# Reactive Hypoglycaemia at Glucose Tolerance Test—Another Presentation of Gestational Diabetes: A Multicentre Retrospective Study

**DOI:** 10.1111/1471-0528.18105

**Published:** 2025-02-25

**Authors:** Sana Rehman, Salsabeel Kazi, Haashim Shah, Nicola Wallis, Pradhiki Mahindra, Bassel H. Al Wattar, Melissa Whitten, Katherine Lattey, Molly Parrington, Rose Turner, Christy Burden, Dimitrios Siassakos

**Affiliations:** ^1^ EGA Institute for Women's Health University College London London UK; ^2^ Cornell University Ithaca, New York USA; ^3^ Comprehensive Clinical Trials Unit, Institute of Clinical Trials and Methodology University College London London UK; ^4^ Beginnings Assisted Conception Unit Epsom and St Helier University Hospitals London UK; ^5^ University College London Hospital NHS Trust London UK; ^6^ University of Bristol Bristol UK; ^7^ Department of Obstetrics and Gynaecology University College London London UK; ^8^ Department of Obstetrics University College London Hospital London UK

**Keywords:** obstetric outcomes, oral glucose tolerance test, reactive hypoglycaemia

## Abstract

**Objective:**

To assess obstetric outcomes in pregnant women with ‘reactive hypoglycaemia’ (RH) during an oral glucose tolerance test (OGTT), defined as a 2‐h blood glucose level lower than the fasting value.

**Design:**

Retrospective observational study.

**Setting:**

Two tertiary maternity units in the United Kingdom.

**Population:**

A total of 1498 women with singleton pregnancies attending for an OGTT between April 2019 and July 2020.

**Methods:**

Maternal and neonatal outcomes were compared between three groups: gestational diabetes, reactive hypoglycaemia and normal OGTT. Both logistic and linear regression models were used, which adjusted for maternal age at booking, ethnicity, parity and BMI.

**Main Outcome Measures:**

Abdominal circumference > 95th centile, polyhydramnios, gestational age at delivery, preterm birth, birthweight, neonatal hypoglycaemia, admission to neonatal unit, perinatal mortality.

**Results:**

Of the 1498 women, 26.7% (*n* = 400) had reactive hypoglycaemia, 27.8% (*n* = 417) GDM and 45.4% (*n* = 681) normal GTT. The reactive hypoglycaemia group were twice as likely to develop polyhydramnios compared with both the GDM (OR 2.77, 95% CI 1.40–5.50) and control groups (OR 2.47, 95% CI 1.31–4.65). Relative to those with GDM, women with reactive hypoglycaemia had a similar mean birthweight (mean difference 59.4 g, *p* = 0.06) but were less likely to experience neonatal hypoglycaemia (OR 0.30, 95% CI 0.001–0.15) or preterm birth (OR 0.33, 95% CI 0.18–0.60). No differences were found in maternal hypertensive disorders, abdominal circumference > 95th centile, shoulder dystocia, Apgar < 7, cord pH, admission to neonatal unit or perinatal mortality.

**Conclusion:**

Women with reactive hypoglycaemia in this sample were at risk of adverse outcomes frequently associated with diabetes, including polyhydramnios.

## 1 Introduction

In the United Kingdom, at least 1:20 pregnancies are affected by gestational diabetes mellitus (GDM), and the prevalence is set to increase, reflecting increasing obesity and maternal age [1, 2]. Women with GDM are at greater risk of adverse outcomes including shoulder dystocia and stillbirth [1, 3]. Effective management in pregnancy greatly reduces these risks [4, 5]. Hence, sensitive screening for GDM is key to enable timely management and optimisation of outcomes. Women at risk of GDM but not screened have a 44% greater risk of stillbirth compared with low‐risk women (aOR 1.44, 95% CI 1.01–2.06) [6].

The optimal screening and testing approach for GDM remains controversial. In the United Kingdom, as per NICE guidelines, women at risk of GDM are offered screening with a 2‐h 75 g oral glucose tolerance test (GTT) [1]. However, the recommended thresholds may not be sensitive enough to detect all forms of glucose dysregulation [7]. A study of 237 pregnancies in women with abnormal placental histopathology found that all women with delayed villous maturation, a pathology strongly associated with diabetes, had normal blood pressure, uterine dopplers and PAPP‐A levels, and none had formal diagnosis of GDM. One in five experienced an ‘unexplained’ stillbirth [8]. Underdiagnosis of glucose dysregulation creates a missed opportunity for intervention.

A proportion of women have ‘reactive hypoglycaemia’ (RH) during pregnancy, a blood glucose level lower after a glucose load compared with the fasting level. There has been limited research into its significance, with varying definitions. A systematic review found that RH is associated with a greater risk of small‐for‐gestational age infants and polyhydramnios [9]. A proposed mechanism is that impaired first‐phase insulin response leads to higher circulating glucose, with subsequent excessive secretion of insulin in the second phase and in turn postprandial hypoglycaemia [10].

At present, RH is not consistently considered an abnormal test result and has no influence on pregnancy care. However, in a specialist quaternary clinic for women with previous adverse pregnancy outcomes at University College London Hospital (UCLH), many are found retrospectively to have had RH using a less restrictive definition of hypoglycaemia, which includes any value at 2 h lower than fasting. In previous pregnancies, these women had experienced a wider range of adverse outcomes, including perinatal death, placental abruption, and neonatal stroke. The aim of this study was to evaluate and characterise obstetric outcomes in women with such broadly defined RH.

## 2 Material and Methods

### 2.1 Study Design and Setting

This was a retrospective study of pregnant women attending for GTT at University College London Hospital, a large tertiary referral unit in central London, and Southmead Hospital, a tertiary referral unit in Bristol, between April 2019 and July 2020. This was a clinical retrospective data analysis of existing databases with no woman identifiable data. Ethics committee approval was not required.

### 2.2 Participants

Electronic health records were used to identify women who had a GTT within the specified period. Women were screened with a GTT if they had at least one of the high‐risk NICE criteria: BMI > 30 kg/m2, previous macrosomic baby (≥ 4.5 kg), previous GDM, first‐degree relative with diabetes or South Asian, Black Caribbean or Middle Eastern ethnicity [1]. Women were then grouped according to their GTT result, as GDM, RH or ‘normal’ GTT (controls). RH was defined as any 2‐h blood glucose measurement less than or equivalent to the fasting value. Diagnosis of GDM at UCLH was based on modified criteria that used the following thresholds: blood glucose > 5.6 mmol/L when fasting; > 10.5 mmol/L at 1 h or > 7.8 mmol/L and 2 h following a 75 g glucose load. In Bristol, the diagnosis of GDM was based on conventional NICE criteria [1]. GTT results that did not fulfil either RH or GDM criteria were considered as controls in both hospitals. Women were excluded from analysis if they had no outcome data available, born elsewhere, had a multiple pregnancy or gestational age at delivery < 28 weeks. Women with RH were identified retrospectively. They had received identical care to controls in both hospitals.

A total of 2576 pregnancies at UCLH had a full GTT. Of these, 14.2% had RH (*n* = 365), 65.6% had a normal GTT (*n* = 1691), and 20.2% had GDM (*n* = 520). As there were far more women who were in the control group who had a GTT, to make the groups comparable in size, we selected a consecutive sample of women (controls, GDM) to collect data on. Following this and further exclusions for missing outcomes, 974 women had corresponding outcome data (Figure S1). Of these, 31.2% (*n* = 304) had RH, 30.7% (*n* = 299) had normal GTT, and 38.1% (*n* = 371) had GDM. Thirty women who had initial RH went on to have diagnosis and management of GDM after 28 weeks and were included in the GDM group for analysis. Ten women had both RH and GDM, and these were managed as GDM and were also included in the GDM group for analysis. A total of 730 pregnancies from Bristol Southmead Hospital had a full GTT result available. Following exclusions, 524 women had corresponding outcome data. Of these, 18.3% (*n* = 96) had RH, 72.9% (*n* = 382) had normal GTT, and 8.8% (*n* = 46) had GDM.

### 2.3 Outcomes

Electronic records were reviewed retrospectively to gather outcome data. Demographic data were collected to establish rates of potential confounding factors. This included maternal age at booking, parity, smoking status, body mass index (BMI), ethnicity (self‐reported at booking) and pre‐existing hypertensive disorder. The antenatal outcomes studied included: gestational hypertensive disorder, findings on routine ultrasound performed between 18 + 0 and20 + 6 weeks, including abdominal circumference > 95th centile (based on national averages) and polyhydramnios (defined by amniotic fluid index > 21 cm or deepest vertical pool > 8 cm). The birth outcomes assessed included: gestational age at birth, preterm birth (< 37 weeks), requirement of induction of labour, mode of birth and shoulder dystocia. Fetal and neonatal outcomes studied included: birthweight, cord blood pH < 7.2 (arterial and venous), Apgar score < 7, requirement of specialist neonatal care input (admission to neonatal unit or ‘transitional neonatal care’), neonatal hypoglycaemia (2 prefeed blood sugars < 2 mmol/L or. single reading < 1 mmol/L at any time using blood gas) and perinatal mortality.

### 2.4 Statistical Analysis

Outcome data from the three cohorts were compared. Descriptive statistics were used for demographic data. Continuous variables were analysed using an ANOVA with a post hoc Bonferroni correction whilst categorical variables were analysed using the chi‐squared test. A *p* value < 0.05 was deemed significant for all statistical tests (with the exception of ANOVA where the Bonferroni‐adjusted significance level was 0.017). Both logistic and linear regression models were created for binary and continuous variables, respectively, and adjusted for maternal age at booking, ethnicity, parity and BMI. Smoking was initially included as a covariate; however, the low event rate resulted in convergence issues and was excluded. Statistical analysis was conducted using Python (version 3.11.6) with the aid statistical packages, scipy (version 1.11.2) and statsmodels (version 0.14.0).

## 3 Results

A total of 1498 participants were included in the study, of which 26.7% (*n* = 400) had RH, 27.8% (*n* = 417) GDM, and 45.4% (*n* = 681) normal GTT.

### 3.1 Maternal Demographics

Maternal demographic data for both UCLH and Southmead hospital are summarised in Table 1. The RH group had a lower mean maternal BMI (26.8 kg/m^2^) than both the GDM (27.1 kg/m^2^) and control groups (27.6 kg/m^2^). The RH group also had the double the proportion of women with a BMI < 18.5 kg/m^2^ (3.5% vs. GDM 1.4% vs. controls 1.8%). There was a higher proportion of women who identified as Black British or Other Black background in the GDM group (15%) compared with the RH (5%) and control groups (6%). Similarly, there were a higher proportion of women who identified as Asian British or Other Asian background in the GDM group (25%) compared with the control (16%) and RH groups (11%). There were similar proportions of women who identified as from a White background in the RH (69%) and control (68%) groups. Rates of smoking were lowest amongst women in the GDM group, however, were similar in the RH and control groups (8%). The mean parity and rates of essential hypertension were similar and low between all three groups.

**TABLE 1:** Baseline maternal characteristics at UCLH and Southmead Hospital combined.

Characteristic	RH	GDM	Controls
	*N* = 400	*N* = 417	*N* = 681
Mean maternal age (years)	33.3 ± 5.7	33.8 ± 5.6	32.1 ± 5.6
Mean BMI (kg/m^2^)	26.8 ± 6.6	27.1 ± 6.1	27.6 ± 6.2
< 18.5—no. (%)	14 (3.5)	6 (1.4)	12 (1.8)
18.5–24.9—no. (%)	187 (46.8)	171 (41.0)	259 (38.0)
25–29.9—no. (%)	806 (21.5)	113 (27.1)	157 (23.1)
30–34.9—no. (%)	65 (16.3)	77 (18.5)	165 (24.2)
35–39.9—no. (%)	26 (6.5)	33 (7.9)	61 (9.0)
> 40—no. (%)	22 (5.5)	15 (3.6)	15 (2.2)
Maternal Ethnicity—no. (%)			
White British or other White background	277 (69.3)	199 (47.7)	466 (68.4)
Black British or other Black background	22 (5.5)	61 (14.6)	39 (5.7)
Asian British or other Asian background	46 (11.5)	103 (24.7)	112 (16.4)
Mixed ethnicity	22 (5.5)	15 (3.6)	30 (4.4)
Other ethnic group or not stated	33 (8.3)	39 (9.4)	34 (5.0)
Smoking in current pregnancy	34 (8.5)	10 (2.4)	52 (7.6)
Essential (pre‐pregnancy) hypertension	9 (2.3)	12 (2.9)	15 (2.2)

The outcome data are presented as two separate analyses: Table 2 demonstrates the UCLH data alone whilst Tables 3 and 4 present both the UCLH and Southmead Hospital datasets combined (Table S1).

**TABLE 2:** Univariable analysis of antenatal, birth, fetal and neonatal outcomes at UCLH.

Outcome	RH	GDM	Controls		Odds ratio (95% confidence interval)		
	*N* = 304	*N* = 371	*N* = 299		RH versus controls	GDM versus controls	RH versus GDM
Maternal hypertensive disorder—no. (%)	25 (8.3)	28 (7.5)	24 (8.0)				
Pregnancy induced hypertension	11 (3.6)	8 (2.2)	5 (1.7)				
Pre‐eclampsia	10 (3.3)	11 (3.0)	9 (3.0)				
Borderline hypertension	4 (1.3)	9 (2.4)	10 (3.3)				
Ultrasound abnormalities—no. (%)							
Abdominal circumference > 95th centile	48 (15.8)	48 (12.9)	40 (13.4)		1.21 (0.77–1.91)	0.96 (0.61–1.51)	1.26 (0.82–1.94)
Polyhydramnios	29 (9.5)	14 (3.8)	14 (4.7)		2.15 (1.11–4.15)	0.80 (0.37–1.70)	2.69 (1.39–5.19)
Induction of labour—no. (%)	90 (28.6)	149 (40.2)	100 (33.4)		0.84 (0.59–1.18)	1.34 (0.97–1.83)	0.63 (0.45–0.86)
Mode of birth—no. (%)							
Vaginal	185 (60.9)	187 (50.4)	162 (54.1)				
Emergency caesarean	50 (16.4)	105 (28.3)	76 (25.4)				
Elective caesarean	69 (22.7)	79 (21.3)	61 (20.4)				
Preterm birth < 37 weeks—no. (%)	13 (4.3)	38 (10.2)	14 (4.7)		0.91 (0.42–1.97)	2.32 (1.23–4.37)	0.39 (0.20–0.75)
Shoulder dystocia—no. (%)	6 (2.0)	7 (1.9)	10 (3.3)		0.58 (0.21–1.62)	0.56 (0.21–1.48)	1.05 (0.35–3.15)
Apgar < 7—no. (%)							
1 min	18 (5.9)	32 (8.6)	19 (6.4)		0.93 (0.48–1.80)	1.39 (0.77–2.51)	0.67 (0.37–1.21)
5 min	3 (1.0)	6 (1.6)	3 (1.0)		0.98 (0.20–4.91)	1.62 (0.40–6.54)	0.61 (0.15–2.44)
Cord blood pH < 7.2—no. (%)							
Venous	4 (1.3)	6 (1.6)	5 (1.7)		1.04 (0.27–3.96)	1.02 (0.31–3.40)	1.02 (0.28–3.70)
Arterial	15 (4.9)	39 (10.5)	26 (8.7)		1.44 (0.72–2.87)	0.76 (0.44–1.33)	1.89 (0.99–3.61)
Neonatal hypoglycaemia—no. (%)	10 (3.2)	35 (9.4)	7 (2.3)		1.42 (0.53–3.78)	4.35 (1.90–9.93)	0.33 (0.16–0.67)
Admission to neonatal unit—no. (%)	74 (24.3)	96 (29.5)	88 (29.4)		0.77 (0.54–1.11)	0.84 (0.60–1.18)	0.92 (0.65–1.31)
Perinatal mortality—no. (%)	1 (0.3)	4 (1.1)	1 (0.3)		0.98 (0.06–15.8)	3.25 (0.36–29.2)	0.30 (0.03–2.72)
				***F*‐statistic**	***p*** *		
Mean gestational age at birth (weeks)	39.4 ± 1.5	38.6 ± 1.7	39.4 ± 1.5	26.5	0.64	< 0.00001	< 0.00001
Mean birthweight (grams)	3419 ± 508	3180 ± 501	3370 ± 502	20.1	0.23	< 0.00001	< 0.00001

* Bonferroni adjusted significance level for ANOVA omnibus hypothesis for pairwise *p*‐values is 0.05/3 = 0.017.

**TABLE 3:** Univariable analysis of antenatal, birth, fetal and neonatal outcomes at UCLH and Southmead Hospital combined.

Outcome	RH	GDM	Controls		Odds ratio (95% confidence interval)		
	*N* = 400	*N* = 417	*N* = 681		RH versus controls	GDM versus controls	RH versus GDM
Maternal hypertensive disorder—no. (%)	32 (8.0)	32 (7.7)	44 (6.5)				
Pregnancy‐induced hypertension	14 (3.5)	9 (2.2)	12 (1.8)				
Pre‐eclampsia	14 (3.5)	14 (3.4)	22 (3.2)				
Borderline hypertension	4 (1.0)	9 (2.2)	10 (1.5)				
Ultrasound abnormalities—no. (%)							
Abdominal circumference > 95th centile	66 (16.5)	61 (14.6)	108 (15.9)		1.05 (0.75–1.47)	0.91 (0.65–1.28)	1.15 (0.79–1.68)
Polyhydramnios	30 (7.5)	14 (3.4)	17 (2.5)		3.17 (1.72–5.82)	1.36 (0.66–2.78)	2.33 (1.22–4.47)
Induction of labour—no. (%)	138 (34.5)	172 (41.2)	249 (36.6)		0.91 (0.70–1.18)	1.22 (0.95–1.56)	0.75 (0.57–0.99)
Mode of birth—no. (%)							
Vaginal	253 (63.3)	213 (51.1)	413 (60.6)				
Emergency caesarean	72 (18.0)	112 (26.9)	141 (20.7)				
Elective caesarean	77 (19.3)	90 (21.6)	127 (18.6)				
Preterm birth < 37 weeks—no. (%)	16 (4.0)	43 (10.3)	44 (6.5)		0.60 (0.34–1.08)	1.66 (1.08–2.58)	0.36 (0.20–0.65)
Shoulder dystocia—no. (%)	9 (2.3)	7 (1.7)	21 (3.1)		0.72 (0.33–1.60)	0.54 (0.23–1.27)	1.35 (0.50–3.66)
Apgar < 7—no. (%)							
1 min	23.5 (5.8)	36 (8.6)	42 (6.2)		0.92 (0.55–1.57)	1.44 (0.90–2.28)	0.65 (0.38–1.11)
5 min	4 (1.0)	6 (1.4)	6 (0.9)		1.13 (0.32–4.04)	1.63 (0.52–5.11)	0.69 (0.19–2.47)
Cord blood pH < 7.2—no. (%)							
Venous	9 (2.3)	8 (1.9)	11 (1.6)		0.73 (0.30–1.81)	1.03 (0.41–2.60)	0.71 (0.27–1.89)
Arterial	26 (6.5)	46 (11.0)	61 (9.0)		1.58 (0.94–2.64)	1.03 (0.66–1.60)	1.53 (0.89–2.62)
Neonatal hypoglycaemia—no. (%)	10 (2.5)	35 (8.3)	9 (1.3)		1.91 (0.77–4.75)	6.84 (3.25–14.4)	0.28 (0.14–0.57)
Admission to neonatal unit—no. (%)	84 (21.0)	99 (23.7)	112 (16.4)		1.35 (0.99–1.85)	1.58 (1.17–2.14)	0.85 (0.61–1.19)
Perinatal mortality—no. (%)	1 (0.3)	4 (1.0)	2 (0.3)		0.85 (0.08–9.41)	3.29 (0.60–18.0)	0.26 (0.03–2.33)
				***F*‐statistic**	***p*** *		
Mean gestational age at birth (weeks)	39.4 ± 1.5	38.6 ± 1.7	39.6 ± 1.6	32.9	0.34	< 0.00001	< 0.00001
Mean birthweight (grams)	3408 ± 497	3182 ± 543	3387 ± 499	26.3	0.48	< 0.00001	< 0.00001

* Bonferroni adjusted significance level for ANOVA omnibus hypothesis for pairwise *p* values is 0.05/3 = 0.017.

**TABLE 4:** Multivariable analysis of antenatal, birth, fetal and neonatal outcomes for UCLH and Southmead Hospital combined, adjusted for maternal age, ethnicity, BMI and parity.

Outcome	Adjusted odds ratio (95% CI)		
	RH versus controls	GDM versus controls	RH versus GDM
Abdominal circumference > 95th centile	1.07 (0.76 to 1.53)	1.06 (0.73 to 1.54)	1.04 (0.70 to 1.56)
Polyhydramnios	2.47 (1.31 to 4.65)	0.93 (0.44 to 2.01)	2.77 (1.40 to 5.50)
Induction of labour	1.03 (0.79 to 1.35)	1.31 (0.99 to 1.73)	0.75 (0.55 to 1.02)
Preterm birth < 37 weeks	0.65 (0.36 to 1.19)	2.23 (1.37 to 3.63)	0.33 (0.18 to 0.60)
Shoulder dystocia	0.70 (0.30 to 1.61)	0.55 (0.21 to 1.42)	1.13 (0.40 to 3.20)
Apgar < 7 at 1 min	0.98 (0.57 to 1.69)	1.55 (0.94 to 2.56)	0.60 (0.34 to 1.05)
Admission to neonatal unit	1.06 (0.76 to 1.48)	1.12 (0.81 to 1.56)	0.90 (0.63 to 1.27)
Neonatal hypoglycaemia	1.88 (0.75 to 4.72)	6.27 (2.92 to 13.4)	0.30 (0.001 to 0.15)
	Mean difference (95% CI, *p*‐value)		
Birthweight adjusted for gestational age (grams)	−1.14 (−51.8 to 49.5, *p* = 0.96)	−45.4 (−98.6 to 7.72, *p* = 0.09)	59.4 (−3.23 to 122.1, *p* = 0.06)
Gestational age at birth (weeks)	0.1 (−0.1 to 0.3, *p* = 0.1)	−0.8 (−0.9 to −0.6, *p* < 0.00001)	0.8 (0.6 to 1.0, *p* < 0.00001)

### 3.2 Antenatal Outcomes

At UCLH, women with RH were more than twice as likely to develop polyhydramnios compared with both the GDM (9.5% vs. 3.8%; OR 2.69, 95% CI 1.39–5.19) and control groups (9.5% vs. 4.7%; OR 2.15, 95% CI 1.11–4.15). This association persisted in the combined data set following adjustments for maternal age, ethnicity, BMI and parity (RH vs. controls aOR 2.47, 95% CI 1.31–4.65; RH vs. GDM aOR 2.77, 95% CI 1.40–5.50). An abdominal circumference measuring > 95th centile was similar in the RH group in both the UCLH and combined data set (RH vs. controls aOR 1.07, 95% CI 0.76–1.53; RH vs. GDM aOR 1.04, 95% CI 0.70–1.56).

### 3.3 Birth Outcomes

Mean gestational age at birth was the same between the RH and control groups at UCLH (39.4 ± 1.5 weeks). This was significantly later than the GDM group (38.6 ± 1.7 weeks, *p* < 0.00001). This association persisted in the combined data set following adjustments (RH vs. GDM mean difference 0.8, *p* < 0.00001; GDM v controls mean difference − 0.8, *p* < 0.00001). Women with RH at UCLH were less likely to experience preterm birth than those with GDM (4.3% vs. 10.2%; OR 0.39, 95% CI 0.20–0.75). These rates were similar in the combined data set and the association persisted following adjustments (aOR 0.33, 95% CI 0.18–0.60).

The RH group at UCLH were less likely to have had induction of labour than the GDM group (28.6% vs. 40.2%; OR 0.63, 95% CI 0.45–0.86) This association persisted in the combined data set following adjustments (aOR 0.69, 95% CI 0.49–0.97). Rates of shoulder dystocia were low, and no significant differences were reported across groups in either the UCLH or combined data sets (Tables 2 and 3).

### 3.4 Fetal and Neonatal Outcomes

Mean birthweight was greatest in those with RH (3419 ± 508 g) than with the GDM (3180 ± 501 g, *p* < 0.00001) and control groups (3370 ± 502 g, *p* = 0.23) at UCLH. However, once adjusted for gestational age at delivery in the combined data set, the mean birthweight in the RH group was similar to those with GDM (RH vs. GDM mean difference 59.4 g, *p* = 0.06). Compared to those with GDM, the RH group were less likely to have neonatal hypoglycaemia at UCLH (3.2% vs. 9.4%; OR 0.33, 95% CI 0.16–0.67). This association persisted in the combined data set after adjustments (aOR 0.3, 95% CI 0.001–0.15). In the combined data set, the RH group had a higher rate of neonatal admission compared to the controls (21% vs. 16.4%; OR 1.35, 95% CI 0.99–1.85); however, this did not reach significance. There were no differences in the other outcomes (Tables 2 and 3).

## 4 Discussion

### 4.1 Main Findings

There are two principal findings of this study. First, RH was associated with higher rates of polyhydramnios than GDM and controls. Second, the RH group had a similar mean birthweight compared to those with GDM in this study after adjusting for gestational age at delivery. These findings remain significant after adjustments for maternal age, BMI, parity and ethnicity. Both polyhydramnios and higher birthweight are known to be more prevalent in GDM.

### 4.2 Strengths and Limitations

To our knowledge, this is the first study to define RH as a 2‐h GTT lower than the fasting value. This definition encompassed a broader range of women experiencing RH in pregnancy, thus enabling an evaluation of obstetric outcomes in a population that has not been previously studied. Moreover, additional outcomes were assessed that have not been reported in existing literature on RH. To limit confounding, adjustments were made for maternal age, BMI, parity and ethnicity. There was good representation of the overall obstetric population as the two hospitals serve a diverse demographic, with both inner‐city populations as well as patients referred from outside the local areas.

A possible limitation is that the study only included women undergoing GTT, known to be higher risk for GDM by fulfilling NICE inclusion criteria, rather than the entire obstetric population. Furthermore, the two centres used different diagnostic criteria for GDM. UCLH used a three‐step approach (blood glucose > 5.6 mmol/L when fasting; > 10.5 mmol/L at 1 h or > 7.8 mmol/L at 2 h following a 75 g glucose load) whilst Southmead Hospital used a two‐step approach in line with conventional NICE criteria (blood glucose > 5.6 mmol/L when fasting; > 7.8 mmol/L at 2 h following a 75 g glucose load) [1]. However, studies have shown that NICE criteria is less sensitive than newer three‐step approaches [7]; thus, it is probable that the controls in the Bristol dataset include many women who would have been classed as GDM at UCLH. Any differences between RH and controls are likely more marked than the results suggest. Furthermore, the retrospective design of this study limits our ability to confirm a causal relationship, but the associations are biologically plausible; glucose dysmetabolism would increase amniotic fluid volume.

### 4.3 Interpretation

A recent systematic review evaluating reactive hypoglycaemia and associated pregnancy outcomes found an increased risk of small‐for‐gestational age infants, low birthweight and neonatal unit admission [9]. The present study suggests that RH, more broadly defined, is associated with polyhydramnios, which is a novel finding. Polyhydramnios is typically associated with higher mean birthweights in the context of glucose dysregulation as opposed to low mean birthweights. A likely explanation for this discrepancy is that existing literature on RH has focussed on low blood sugars using an absolute threshold independent of the fasting value. It is possible that increased insulin sensitivity may drive maternal hypoglycaemia, thus limiting the substrates available for fetal growth [11]. Other studies have suggested hypoglycaemia directly limits placental development, resulting in placental insufficiency and subsequent poor fetal growth [12, 13]. Increased insulin sensitivity would not account for the increased rates of polyhydramnios observed in the RH group in the current study; this outcome is an established complication of GDM, as well as fetal growth restriction. Polyhydramnios, in turn, has been associated with a wide range of adverse outcomes, including perinatal death, another catastrophic complication of diabetes [14]. It is therefore plausible that RH may represent another form of glucose dysmetabolism.

Reactive hypoglycaemia has indeed been suggested as a marker of early insulin resistance and prediabetes outside pregnancy by other authors [15, 16]. One proposed mechanism is via an impaired first‐phase insulin response. Insulin is typically secreted in two phases following a glucose load; a disruption in the first phase leads to higher circulating glucose, with subsequent excessive secretion of insulin in the second phase. Excessive insulin secretion results in a sharper drop in circulating glucose, with hypoglycaemia ensuing postprandially [17]. This abnormal pattern of insulin secretion over time may put women at risk of pancreatic beta‐cell exhaustion and subsequent dysregulated glucose metabolism late in pregnancy.

It is clear from this study that the proportion of women with RH, broadly defined, is significant and its impact on perinatal outcomes warrants additional assessment. Future larger prospective multicentre studies using different GDM thresholds, which include RH are required to determine whether these findings are widely applicable, and whether smaller differences in rarer diabetes‐related outcomes may also exist. We also recommend studies in countries with universal screening for GDM as opposed to risk‐stratified approaches to further establish the true impact of RH. We do not propose all women with RH are immediately prescribed insulin. Encouraging carbohydrate restriction and providing lifestyle advice per GDM guidance may be all that is needed for most women with RH.

## 5 Conclusion

In conclusion, this study suggests that RH during pregnancy is associated with polyhydramnios, an adverse outcome frequently associated with diabetes. Whereas it may be argued whether it represents atypical diabetes or not, it may be difficult to ignore RH and label the result as ‘normal’ in a woman investigated for polyhydramnios.

We propose that RH does likely represent an early form of glucose dysregulation and evolving insulin resistance, not insulin hypersensitivity as suggested by the existing literature. Clinicians should acknowledge that the result is not entirely normal; once RH has been identified on a GTT, additional vigilance for complications in this population may bring us one step closer to reducing preventable adverse outcomes related to diabetes.

## Author Contributions

D.S. and C.B. formulated the study and contributed to editing the article. S.R. wrote the article and contributed to data collection. S.K., N.W., K.L., M.P. and R.T. collected data and contributed to editing the article. H.S. and P.M. performed statistical analyses on the data sets and reviewed the final draft. B.H.A.W. and M.W. contributed with planning the study and editing the article. All authors contributed to the intellectual content and study design and approved the final version of this article.

## Ethics Statement

The authors have nothing to report.

## Conflicts of Interest

The authors declare no conflicts of interest.

## Supporting information


Data S1.

## Data Availability

The data that support the findings of this study are available from the corresponding author upon reasonable request.
