# Supplementary material for: Reactive Hypoglycaemia at Glucose Tolerance Test—Another Presentation of Gestational Diabetes: A Multicentre Retrospective Study
Source: BJOG. 2025 Feb 25;132(7):927–34. doi: 10.1111/1471-0528.18105 (PMC12051243; doi:10.1111/1471-0528.18105)
Supplement: Supplementary file 1 — Data S1. [file BJO-132-927-s001.docx]

**Figure S1 –** Flowchart showing inclusion/exclusion of patients at UCLH


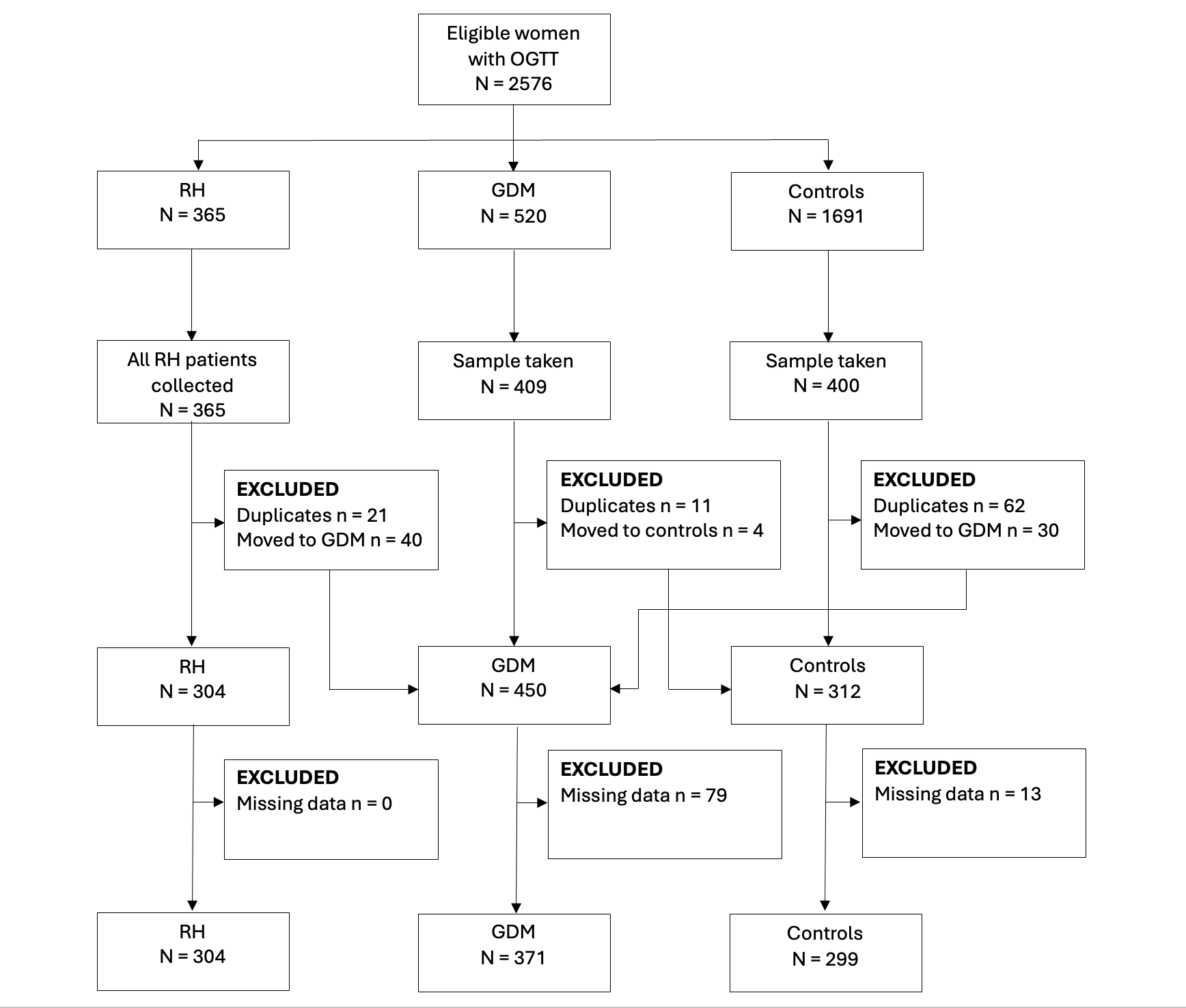


**Table S1 -** Multivariable analysis of antenatal, birth, fetal and neonatal outcomes at UCLH, adjusted for maternal age, ethnicity , BMI and parity

|  | **Adjusted odds ratio (95% CI)** | | |
| --- | --- | --- | --- |
| **Outcome** | **RH v Controls** | **GDM v Controls** | **RH v GDM** |
| Abdominal circumference >95th centile | 1.11 (0.70 - 1.76) | 0.97 (0.61 - 1.54) | 1.16 (0.73 - 1.82) |
| Polyhydramnios | **2.13 (1.09 - 4.16)** | 0.80 (0.37 - 1.74) | **2.79 (1.40 - 5.55)** |
| Induction of labour | 0.86 (0.60 - 1.22) | 1.32 (0.95 - 1.84) | 0.69 (0.49 - 0.97) |
| Preterm birth <37 weeks | 0.98 (0.45 - 2.15) | **2.52 (1.32 - 4.79)** | 0.36 (0.18 - 0.70) |
| Shoulder dystocia | 0.56 (0.19 - 1.63) | 0.56 (0.2 - 1.56) | 0.95 (0.30 - 2.98) |
| Apgar <7 at 1 minute | 0.93 (0.47 - 1.82) | 1.45 (0.80 - 2.63) | 0.60 (0.32 - 1.11) |
| Admission to neonatal unit | 0.80 (0.55 - 1.15) | 0.83 (0.59 - 1.18) | 0.90 (0.63 - 1.30) |
| Neonatal hypoglycaemia | 1.36 (0.50 – 3.68) | **4.29 (1.86 – 9.86)** | **0.35 (0.17 – 0.72)** |
|  |  |  |  |
|  | **Mean difference (95% CI, p-value)** | | |
| Birth weight adjusted by gestational age (grams) | 16.7 (-50.5 – 83.9, p=0.63) | -42.9 (-110.7 – 24.9, p=0.21) | 66.7 (-2.96 – 136.3, p=0.06) |
| Gestational age at birth (weeks) | 0.66 (-0.18 - 0.29, p=0.05) | **-0.74 (-0.99 - -0.50, p<0.00001)** | **0.8 (0.55 - 1.06, p<0.00001)** |
